# Clinical characteristics and prognostic factors affecting survival after radical radiotherapy for early and late post-treatment metastatic nasopharyngeal carcinoma

**DOI:** 10.1186/s12885-022-10494-7

**Published:** 2023-01-03

**Authors:** Guo-Dong Jia, Xue-Song Sun, Xiao-Yun Li, Sai-Lan Liu, Jin-Hao Yang, Qiu-Yan Chen, Li Yuan, Hai-Qiang Mai

**Affiliations:** 1 Sun Yat-sen University Cancer Center, State Key Laboratory of Oncology in South China, Collaborative Innovation Center for Cancer Medicine, Guangdong Key Laboratory of Nasopharyngeal Carcinoma Diagnosis and Therapy, Guangzhou, Guangdong Province People’s Republic of China; 2 Department of Nasopharyngeal Carcinoma, Sun Yat-sen University Cancer Center, Guangzhou, Guangdong Province People’s Republic of China

**Keywords:** Nasopharyngeal carcinoma, Prognosis, Early metastatic, Late metastatic

## Abstract

**Objective:**

We compared the clinical characteristics and survival outcomes after radical radiotherapy between nasopharyngeal carcinoma (NPC) with early and late metastases based on a relatively large cohort, which provides valuable data for the planning of clinical surveillance strategies.

**Methods:**

This was a single-center retrospective analysis of 10,566 patients who received radical radiotherapy in China from January 2000 to December 2016. Overall survival was the primary endpoint. Kaplan–Meier survival analysis and log-rank tests were applied to investigate the association between early or late metastasis and the endpoints. The prognostic value of clinicopathological features was identified using univariate and multivariate Cox proportional hazards models.

**Results:**

The cutoff value for time to metastasis was based on ROC analysis. A total of 559 (5.3%) patients developed distant metastases, 297 (53.1%) of which developed early metastatic disease, with the rest (46.9%) developing late metastatic disease. The K-M analysis showed that the patients with late metastatic foci had significantly better post-metastatic OS (*P* = 0.0056). Multivariate analysis indicated that age, liver metastasis, the number of metastatic foci and time to metastasis (*P* = 0.013) are independent prognostic factors for OS. After analyzing the impact of different treatment methods, we found that local treatment was an independent protective factor for LM, while local treatment was not associated with a survival benefit for EM disease.

**Conclusions:**

The time to metastasis after radical radiotherapy affected the prognosis of NPC patients and local treatment was an independent protective factor that could improve the survival of late metastatic NPC patients.

**Supplementary Information:**

The online version contains supplementary material available at 10.1186/s12885-022-10494-7.

## Introduction

Nasopharyngeal carcinoma (NPC), a malignant tumor that originates in the nasopharyngeal epithelium, is endemic in Southern China, Southeast Asia, North Africa, the Middle East, and Alaska [1, 2]. As a result of its complex anatomical location and high radio-sensitivity, radiotherapy with or without chemotherapy is the primary treatment modality for NPC [3]. The application of intensity modulated radiation therapy (IMRT) has greatly improved locoregional control [4], and distant metastasis has become the main cause of treatment failure in NPC patients [5].

Many pathological mechanisms are involved in distant metastasis [6–8]. Reasons for failure may include the presence of undetected micrometastases, a large initial tumor burden, poor drug access to tumor cells, primary drug resistance, and tumor dormancy-reactivation [9–14]. Evidence suggests that in many cases, tumor cells have already seeded metastatic sites by the time the primary tumor is detected [11, 13, 14]. Dormant cells are activated at different times to form new metastatic foci. The existence of these above mechanisms may affect the time of tumor metastasis and the biological characteristics of tumor cells, including tumor invasiveness. Therefore, the different time of metastasis in patients with NPC may also have an impact on their survival.

To the best of our knowledge, research focused on the time to NPC metastasis remains rare and limited. In the current study, we focused on individual differences in the time to metastasis in NPC patients. All patients were divided into early metastatic (EM) and late metastatic (LM) subgroups. Finally, we also investigated the prognostic value of the time interval between radiotherapy and disease progression, which provides valuable data for the planning of clinical surveillance strategies.

## Patients and methods

### Patient selection

**Fig. 1:**
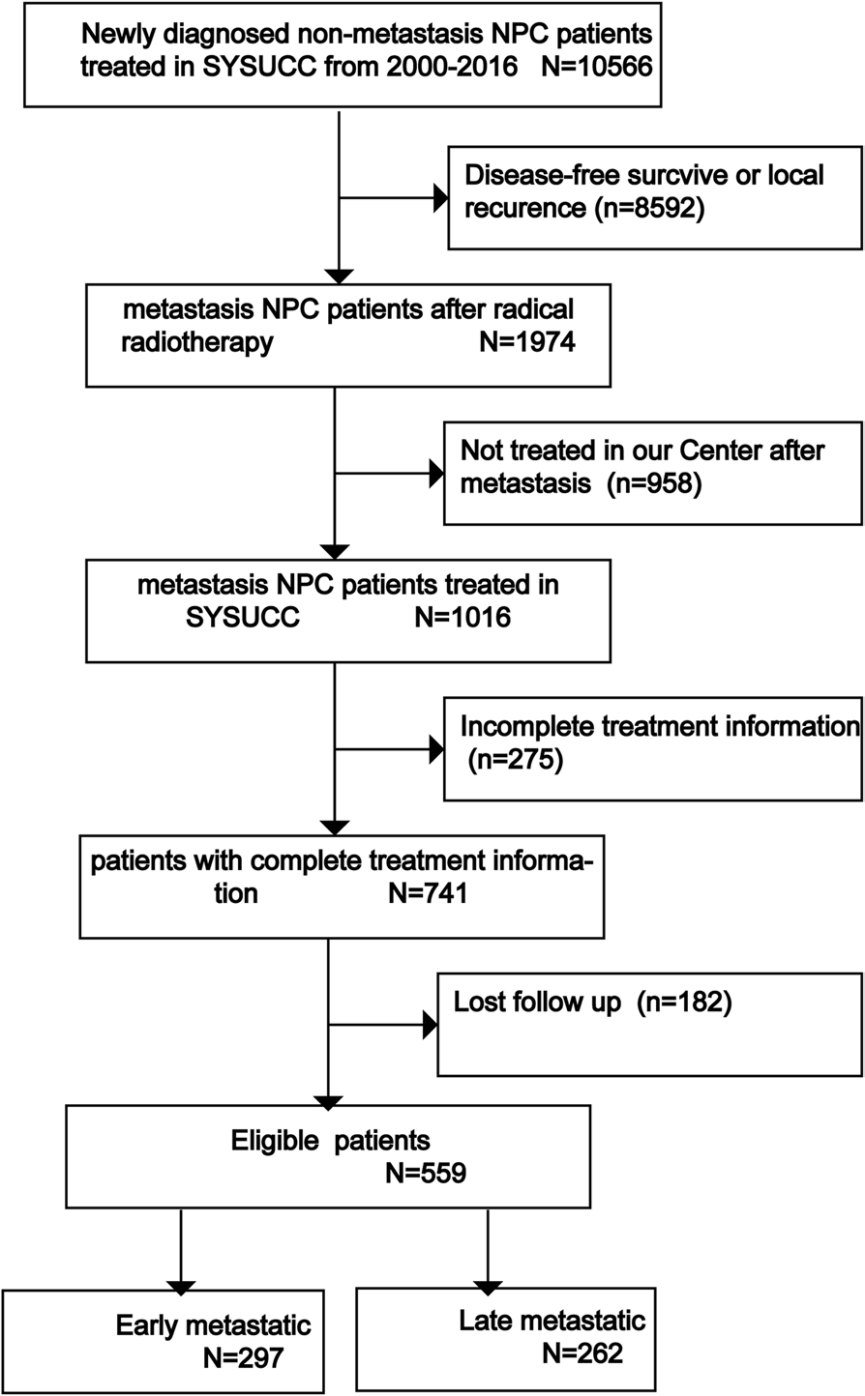
Flow diagram of the patient selection process. Inclusion and exclusion criteria

We retrospectively reviewed the records of 10,566 patients with pathohistologically confirmed NPC (identified using the International Classification of Diseases for Oncology (ICD-O) code ‘C11’), who received IMRT at our center between January 2000 and December 2016. Finally, 559 patients were included in this study. The pathological types included differentiated non-keratinizing carcinoma (World Health Organization [WHO] type II; ICD-O histology codes 8072 and 8073) and undifferentiated non-keratinizing carcinoma (WHO type III; ICD-O histology codes 8020, 8021, and 8082) [15]. Our eligibility criteria included: M0 stage at the time of diagnosis; achieved complete response (CR) or partial response (PR) after primary treatment; distant lesions occurred after initial treatment; no history of other malignancies; satisfactory liver and kidney function; complete treatment information and histologically confirmed NPC. The exclusion criteria were as follows: metastasis at the time of diagnosis, recurrent NPC patients, patients with insufficient clinical data. All patients were restaged according to the 8th Union for International Cancer Control/American Joint Committee on Cancer staging system [16, 17]. This study was performed according to the ethical principles of the Declaration of Helsinki, and the Sun Yat-Sen University Cancer Center review board approved the study protocol. Written informed consent was obtained from all patients for their data to be used in clinical research without affecting their treatment options or violating their privacy. A flowchart illustrating the patient inclusion process is shown in Fig. 1. All patients had completed a pretreatment evaluation including complete patient history, physical examination, hematology and biochemistry profiles, magnetic resonance imaging (MRI) of the nasopharynx and neck, chest radiography, abdominal ultrasonography and whole-body bone scan or positron emission tomography/computed tomography (PET/CT).

### Treatment

All patients received palliative chemotherapy (PCT). The common PCT regimens were listed as follows: PF (20–30 mg/m^2^ cisplatin intravenously [IV] on days 1–3 plus 800–1000 mg/m^2^ 5-fluorouracil continuous IV infusion for 24 h on days 1–5), TP (75 mg/m^2^ docetaxel IV on day 1 plus 20–25 mg/m^2^ cisplatin IV on days 1–3), TPF (60 mg/m^2^ docetaxel IV on day 1 plus 20–25 mg/m^2^ cisplatin IV on days 1–3 plus 500–800 mg/m^2^ 5-fluorouracil continuous IV infusion for 24 h on days 1–5), GP (20–30 mg/m^2^ cisplatin IV on days 1–3 plus 800–1000 mg/m^2^ gemcitabine IV on days 1 and 8). Chemotherapy was intravenously administered at 3-week intervals and the median cycle of PCT was four (range: 2–10 cycles). A total of 138 patients (24.7%) received local treatment of metastasis combined with PCT. Radiotherapy was applied to the metastatic site using two-dimensional conventional radiotherapy (2D-CRT), intensity-modulated radiotherapy (IMRT) or stereotactic body radiation therapy (SBRT). The accumulated radiation dose was 30–50 Gy, 5 times a week, at approximately 2 Gy per fraction for 2D-CRT and IMRT, and 7 Gy per fraction for SBRT.

### Follow-up schedule and definition of EM and LM

Patients attended follow-up visits every 3 months during the first 2 years, every 6 months during years 3–5, and annually thereafter or until death. Post-treatment metastasis was diagnosed by pathological examination via fine-needle aspiration or surgery, or by radiology with local magnetic resonance imaging (MRI) with contrast, nasopharyngoscopy, chest radiography/computed tomography (CT) with contrast, abdominal sonography/CT with contrast, electrocardiography, or bone scans, where appropriate. Positron emission tomography computed tomography (PET-CT) was also used as an alternative diagnostic modality. The primary endpoint of the study was overall survival from the time point of primary diagnosis of metastatic disease.

### Statistical analysis

All patients’ characteristics were converted into categorical variables. The cutoff value of age was based on the median of the entire cohort. The cutoff value of number of metastatic lesions was based on a previous study [18]. The cohort was divided into EM and LM subgroups based on the cutoff value of the time to metastasis, which was determined using a receiver operating characteristic (ROC) curve. The clinicopathological characteristics and treatment modalities among the patients with EM and LM were compared using the chi-squared test. The post-metastatic OS was calculated using the Kaplan–Meier method, and differences between survival curves were assessed using the log-rank test. The prognostic factors associated with the post-metastatic OS of patients with EM and LM disease were evaluated using multivariate Cox regression analysis. Differences with two-sided *P*-values < 0.05 were considered statistically significant. Statistical analyses were performed using SPSS version 23.0 (IBM Corp., USA).

## Results

### Comparison of clinical characteristics between patients with EM and LM

**Fig. 2:**
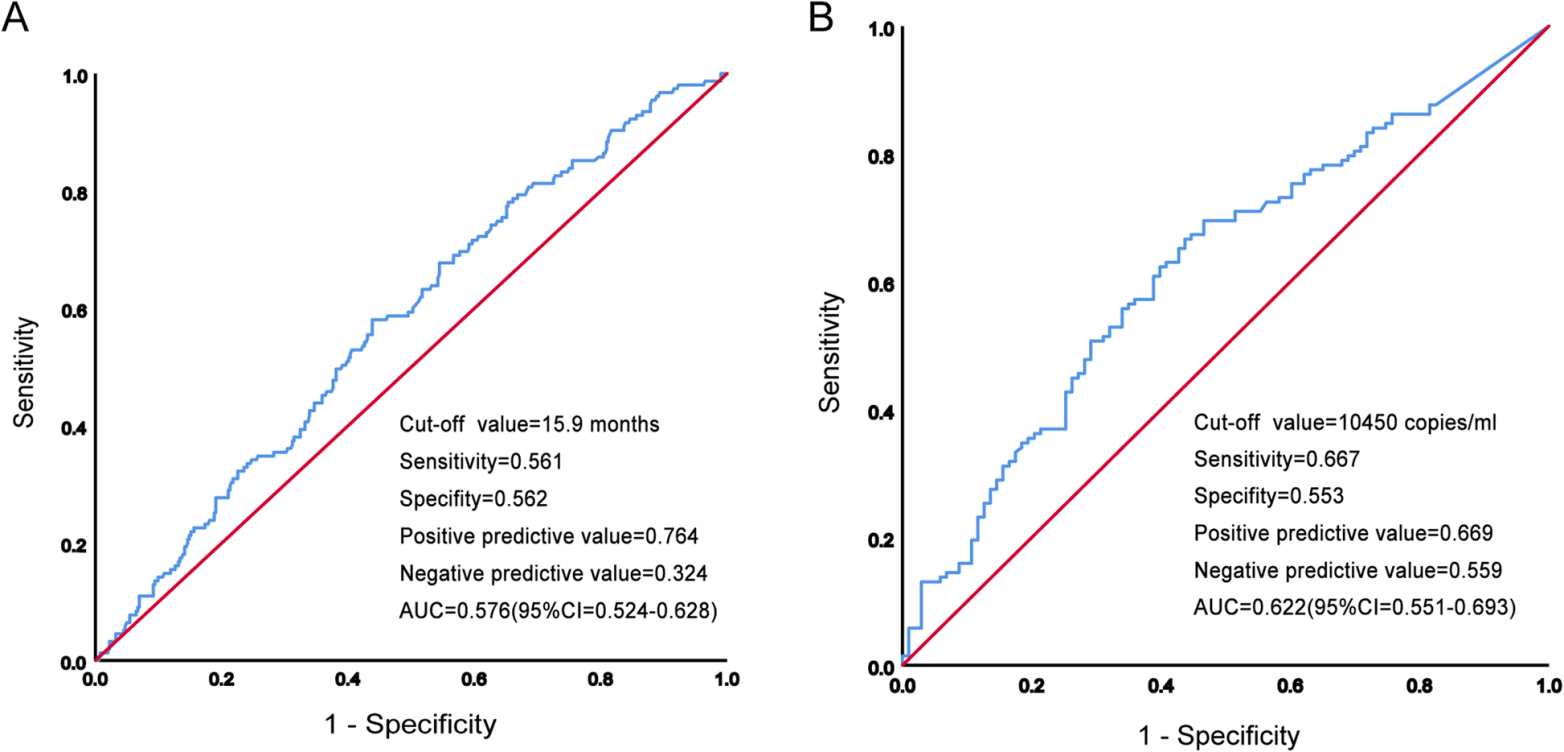
Receiver operating characteristic curves (ROC) for time interval (**A**) and EBV DNA (**B**)

During the median follow up time of 21.7 months (interquartile range [IQR]:13.1–38.8 months), a total of 404 patients (72.3%) died. Among all 559 patients, 447 patients (79.9%) were male, and the median age was 45.0 years. The median time interval between initial radical radiotherapy and metastasis in the whole cohort was 15.1 months (interquartile range [IQR]: 9.5–24.6 months). Patients were divided into two groups according to the time interval between initial radical radiotherapy and metastasis (TI), with a cut-off value of 15.9 months based on ROC analysis (Fig. 2A). According to the ROC curve analysis, the cut-off value was 10,450 copies/mL applied to discriminate OS curves of the two groups (sensitivity = 0.667, specificity = 0.553, area under curve [AUC] = 0.622) (Fig. 2B).

**Fig. 3:**
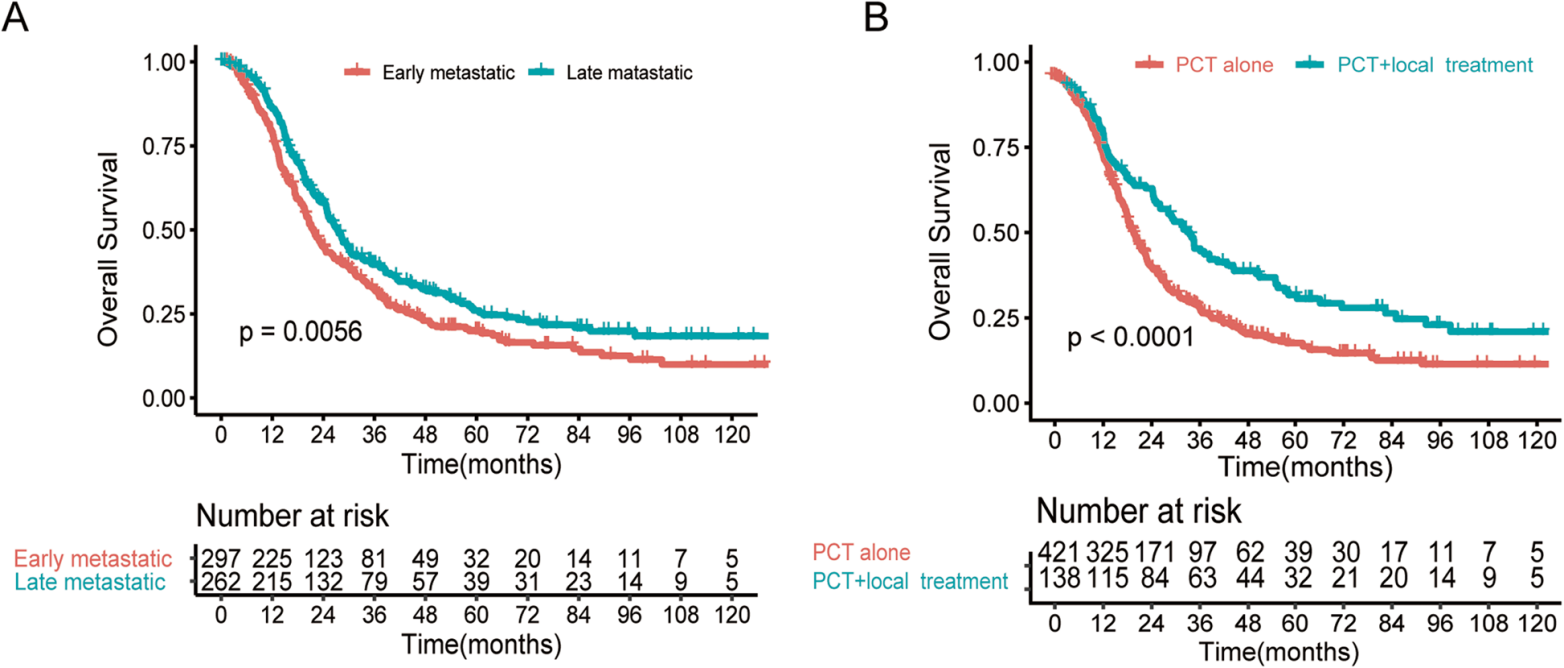
Kaplan–Meier curves for overall survival among groups with TI (**A**), with or without local treatment (**B**)

To optimize the cut- off value for its potential acceptance and clinical application, we rounded to the nearest integer of 10,000 copies/mL (Fig. 2B). Finally, 297 patients were assigned to the EM group and 262 to the LM group. Overall survival at 3 years was 34.0% in the EM group, and 39.8% in the LM group (*p* = 0.0056; Fig. 3A). In terms of treatment method, patients in the PCT + local group achieved higher 3-year OS rate than PCT alone group (51.1% vs.31.1%, *P* < 0.0001; Fig. 3B).

The clinicopathological characteristics of patients in these two groups are shown in Table 1. The site of metastasis was significantly different between LM and EM groups. The proportion of patients with liver and bone metastases was higher in the EM group than in the LM group, while lung metastasis and distant lymph node metastasis occurred more often in the LM group. No significant difference in treatment method was found between the EM and LM groups. The detailed information of multiple organ metastases was shown in Table S1.

**Table 1:** Comparison of clinical characteristics of EM and LM patients with metastatic NPC (*n* = 559)

Characteristic	Total (*n* = 559)	EM group (*n* = 297)	LM group (*n* = 262)	*P*-value
**Gender**				0.752
Female	112(20.0%)	61(20.5%)	51(19.5)	
Male	447(80.0%)	236(79.5%)	211(80.5%)	
**Age (years)**				0.598
≤ 45	284(50.8%)	154(51.9%)	130(49.6%)	
> 45	275(49.2%)	143(48.1%)	132(50.4%)	
**Smoking status**				0.153
Non-smoker	230(56.7%)	109(53.2%)	121(60.2%)	
Smoker	176(43.3%)	96(46.8%)	80(39.8%)	
**Number of metastatic lesions**				0.934
≤ 3	223(39.9%)	118(39.7%)	105(40.1%)	
> 3	336(60.1%)	179(60.3%)	157(59.9%)	
**Baseline value of EBV-DNA**				0.900
≥ 10,000	139(57.7%)	72(58.1%)	67(57.3%)	
< 10,000	102(42.3%)	52(41.9%)	50(42.7%)	
**Bone metastatic**				0.035
Yes	244(43.6%)	142(47.8%)	102(38.9%)	
No	315(56.4%)	155(52.2%)	160(61.1%)	
**Lung metastatic**				0.001
Yes	213(38.1%)	91(30.6%)	122(46.6%)	
No	346(61.9%)	206(69.4%)	140(53.4%)	
**Liver metastatic**				0.018
Yes	208(37.2%)	124(41.8%)	84(32.1%)	
No	351(62.8%)	173(58.2%)	178(67.9%)	
**Distant nodal metastatic**				0.001
Yes	103(18.4%)	38(12.8%)	65(24.8%)	
No	456(81.6%)	259(87.2%)	197(75.2%)	
**Local therapy**				0.692
Yes	138(24.7%)	69(23.2%)	69(26.3%)	
No	421(75.3%)	228(76.8%)	193(73.7%)	

*Abbreviations EBV* Epstein-Barr virus, *EM* early metastatic, *LM* late metastatic

The *P*-values were calculated using Pearson ‘s χ^2^ test

### Prognostic factors associated with post-metastatic OS

Multivariable analysis was performed to screen out potential prognostic factors for OS (Table 2). In multivariate analysis, age (HR, 1.36, 95%CI, 1.11–1.65, *P* = 0.003), sex (HR, 0.77, 95%CI, 0.61–0.98, *P* = 0.033), time to metastasis (HR: 0.78, 95%CI: 0.64–0.95; *P* = 0.014), liver metastasis (HR: 1.66; 95%CI: 1.35–2.03; *P* < 0.001), local treatment of metastasis (HR: 0.72; 95%CI: 0.56–0.91; *P* = 0.007) and the number of metastatic foci (HR: 1.87; 95%CI: 1.51–2.32; *P* < 0.001) were found to be independent prognostic factors for OS.

**Table 2:** Multivariate analysis of prognostic factors for overall survival

Variables	Multivariable	
	**HR (95%CI)**	***P* value**
Gender	0.78(0.61–0.98)	0.035
Age (years)	1.36(1.11–1.65)	0.003
Time to metastasis (years)	0.77(0.63–0.94)	0.013
Number of metastatic lesions	1.84(1.46–2.32)	< 0.001
Bone metastatic	1.24(0.98–1.58)	0.075
Lung metastatic	1.06(0.82–1.36)	0.655
Liver metastatic	1.70(1.35–2.14)	< 0.001
Distant nodal metastatic	0.78(0.80–1.36)	0.780
Local therapy	0.72(0.57–0.92)	0.009

*Abbreviations HR* hazard ratio, *CI* confidence interval

A Cox proportional hazards model was used to perform multivariate analyses. All variables were transformed into categorical variables. HRs were calculated for Gender (Female vs. Male); Age (years) (> 45 vs. ≤ 45); Number of metastases (> 3 vs. ≤ 3); Time to metastasis (months) (> 15.9 vs. ≤ 15.9); Bone metastatic (Yes vs. No); Lung metastatic (Yes vs. No); Liver metastatic (Yes vs. No); Distant nodal metastatic (Yes vs. No); Local therapy (Yes vs. No).

### The role of LT in EM and LM Subgroups

**Fig. 4:**
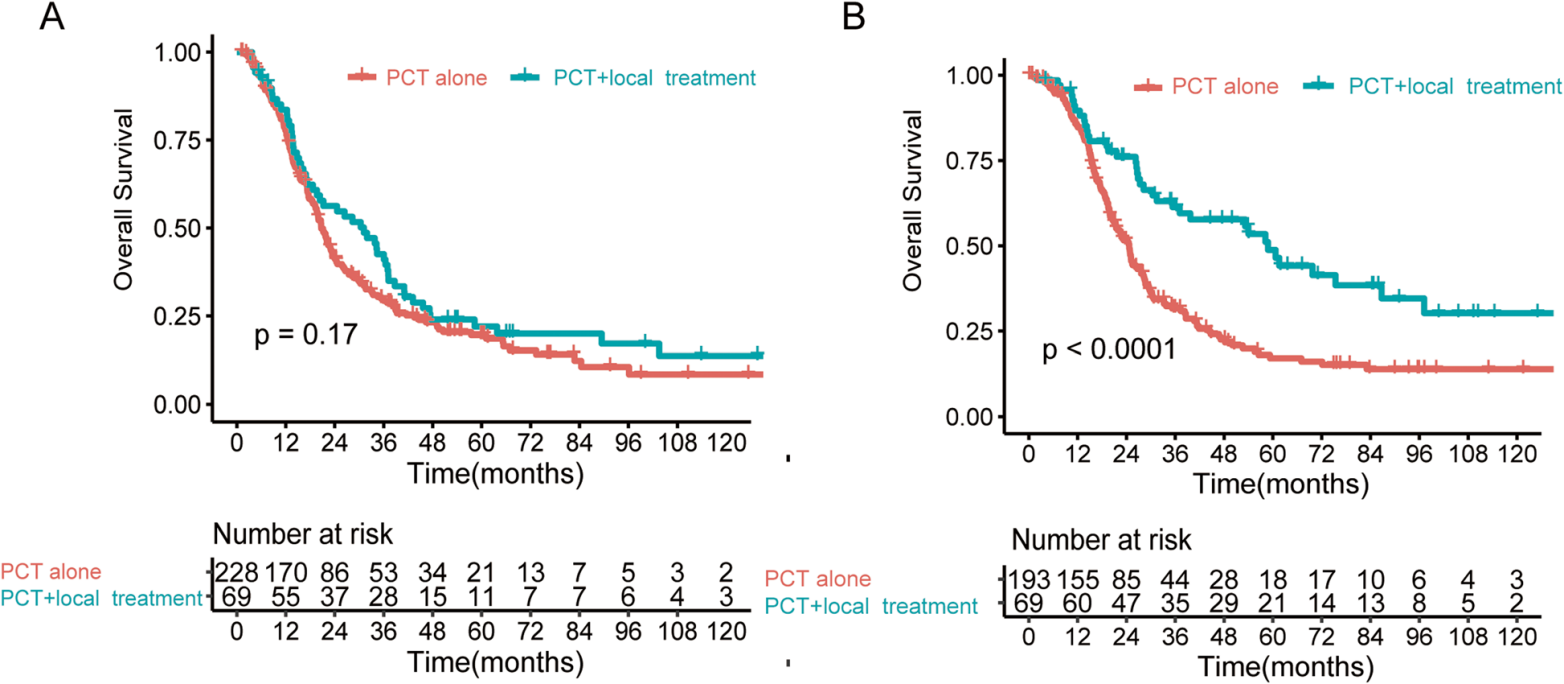
Kaplan–Meier curves for overall survival in subgroups with or without local treatment from the EM group (**A**) and the LM group (**B**), respectively

We further investigated the efficacy of LT in EM and LM patients. Among patients with TI > 15.9 months, the 3-year OS rate of the group without LT was significantly lower than that of the LT group (41.1% vs. 61.3%, *P* < 0.0001) (Fig. 4B). However, among patients with TI ≤ 15.9 months, the 3-year OS rate was comparable in these two groups with or without LT (31.6% vs. 30.7%, *P* = 0.17) (Fig. 4A).

In stratified multivariate analyses (Table 3), Cox regression analysis indicated that age (HR: 1.35; 95%CI: 1.04–1.76; *P* = 0.027), presence of bone metastasis (HR: 1.43; 95%CI: 1.04–1.97; *P* = 0.029), presence of liver metastasis (HR: 2.16; 95%CI: 1.58–2.94; *P* < 0.001) and the number of metastatic foci (HR: 1.72; 95%CI: 1.26–2.34; *P* = 0.001) were independent risk factors for OS among EM patients. In the LM group (*n* = 262), cox regression analysis indicated that age (HR:1.47; 95%CI: 1.08–2.01; *P* = 0.014), local treatment (HR 0.60; 95%CI: 0.41–0.88; *P* = 0.009), and number of metastatic sites (HR: 2.03; 95%CI: 1.41–2.94; *P* < 0.001) were independent risk factors for OS. Therefore, similar to univariate results, local treatment was an independent protective factor for patients only in the LM group.

**Table 3:** Multivariate analysis of OS in EM and LM patients with metastatic NPC

**Characteristic**	**Early metastatic patients**			**Late metastatic patients**		
	**HR**	**95% CI**	***P*-value**	**HR**	**95% CI**	***P*-value**
	0.72	0.52–0.98	0.038	0.84	0.58–1.22	0.356
	1.35	1.04–1.76	0.027	1.47	1.08–2.01	0.014
	1.72	1.26–2.34	0.001	2.03	1.41–2.94	< 0.001
	1.43	1.04–1.97	0.029	1.17	0.81–1.69	0.408
	0.94	0.67–1.33	0.732	1.28	0.87–1.87	0.207
	2.16	1.58–2.94	< 0.001	1.29	0.90–1.84	0.166
	0.97	0.64–1.45	0.871	1.22	0.83–2.78	0.310
	0.84	0.61–1.15	0.269	0.60	0.41–0.88	0.009

*Abbreviations EM* early metastatic, *LM* late metastatic, *HR* hazard ratio, *CI* confidence interval

A Cox proportional hazard model was used to perform multivariate analyses. All variables were transformed into categorical variables. HRs were calculated for Gender (Female vs. Male); Age (years) (> 45 vs. ≤ 45); Number of metastases (> 3 vs. ≤ 3);; Bone metastatic (Yes vs. No); Lung metastatic (Yes vs. No); Liver metastatic (Yes vs. No); Distant nodal metastatic (Yes vs. No); Local therapy (Yes vs. No);

## Discussion

To the best of our knowledge, this is the first study to investigate the influence of the time to metastasis on the prognosis of post-treatment metastatic NPC patients, which were divided into EM and LM groups. In this retrospective study, we found that 297 patients (53.1%) developed early metastatic, and 262 patients (46.9%) developed late metastatic disease, which suggests that the incidence of early and late metastatic nasopharyngeal carcinoma is nearly the same. Additionally, the time to metastasis was found to be an independent prognostic factors for post-metastatic OS and the prognosis of the EM group was worse than that of the LM group, even if its accuracy in predicting patient survival is lower than that of traditional biomarkers (such as EBV DNA). We speculated the reason was that the time factor could not reflect the tumor burden directly, which was also affected by the follow-up strategy and conditions of patients after radiotherapy. Moreover, local treatment had a significant survival benefit only in the LM group, while no benefit was found in the EM group.

Distant metastasis is still the main cause of treatment failure and death in NPC patients [5]. The clinical outcomes of these patients are very poor, with a median overall survival of about 22 months with first-line platinum chemotherapy [19]. Although the overall survival rate is not satisfactory, a small number of patients can nevertheless achieve long-term survival [2]. Due to the heterogeneity of metastatic NPC, the conditions of individual patients are very different, and it is necessary to establish individualized treatment plans.

Whether local treatment of metastatic NPC in addition to traditional palliative chemotherapy is warranted should also be considered. Our previous study showed that patients with metastatic NPC can benefit from local treatment [20]. Furthermore, the NCCN guidelines indicate that limited metastatic NPC has the chance to achieve long-term survival through local treatment, including radiotherapy, surgery or ablation, which may play a role in oligometastatic tumors [21]. Consistently, local treatment of metastasis was identified as a predictor of longer survival in patients with metastatic NPC after treatment in this study. The OS of patients with local treatment was significantly longer than that of patients without local treatment, with a HR of 0.72 (95%CI:0.57–0.92).

However, there is heterogeneity among tumor patients, and it remains unclear if all patients with metastatic disease can benefit from local treatment. There are dormant cells inside tumors, and these cells are activated under certain conditions [14], resulting in aggravated invasion and metastasis. However, the time required for this process varies from individual to individual [13]. The currently popular tumor dormancy-reactivation theory states that in many cases, tumor cells have already been seeded to metastatic sites at the time of diagnosis of the primary tumor. Subsequently, resting cells will be activated at different times to form new metastatic foci [14]. Therefore, the different time of tumor metastasis may be correlated with biological characteristics of tumor cells. At present, only one study investigated the relationship between tumor progression and prognosis in NPC. Zhou and colleagues [22] divided the patients into early and late recurrence groups according to the time to recurrence after radiotherapy. The results showed that the overall survival of patients with early recurrence was significantly longer than that of patients with late recurrence (*p* < 0.001). Nevertheless, we are not aware of any relevant reports investigating the time of metastasis. In this study, we compared the survival of patients with metastases appearing at different times and found that patients in the LM group with an HR of 0.77 (95% CI: 0.63–0.94) achieved significantly higher 3-year OS compared with patients in the EM group (39.8 vs. 34.0%, *P* = 0.0056).

After exploring the survival effect of local treatment in the LM and EM groups, we found that local treatment has significant survival benefits for patients in the LM group (HR:0.60,95%CI:0.41–0.88, *P* = 0.009), but it did not improve the survival of patients in the EM group (HR:0.84, 95%CI:0.61–1.15, *P* = 0.269). Our results show that patients with advanced metastatic NPC can benefit from local treatment, but local treatment for patients with early metastasis cannot effectively improve their survival, which may help guide clinical treatment.

Additionally, our study also found a correlation between the timing and target organs of metastasis for the first time. The chi-squared test indicated that liver and bone metastasis was more common in EM group, while lung metastasis mainly occurred in the LM group, which may be related to the early response of specific organs to chemotactic signals [23]. The prognosis of patients with liver metastasis is poor, which is consistent with the results of our multivariate analysis. Therefore, the target organs can also be considered as factors in the prognosis of patients with early metastasis.

With the increasingly better prognosis of NPC in recent years, clinicians are more strongly advocating the screening of patients who can be effectively treated for metastatic tumors, in order to achieve the goal of long-term survival instead of merely palliative local treatment. Screening the population that can benefit most from local treatment based on the time to metastasis can provide a new strategy for clinical individualized treatment. Our results provide a theoretical basis for screening patients with metastatic NPC after radical radiotherapy who can benefit from local treatment of the metastasis. Although our study yielded some novel results, certain limitations should be noted. Firstly, this is a retrospective single-center study, which has inherent biases. There is heterogeneity in the local treatment methods and metastatic target organs within the group. Due to the low incidence of metastatic NPC, it is difficult to analyze subgroups stratified by treatment methods and metastatic target organs. Secondly, information on the presence of EBV DNA in tumor cells has only become available in recent years, and could not be evaluated in this study.

In conclusion, the time to metastasis after radical radiotherapy affected the prognosis of NPC patients, and local treatment was an independent protective factor in late metastatic NPC patients. In the future, a well-designed, multicenter, prospective randomized design combined with biomarker assessment is needed to verify this conclusion.

### Supplementary Information

**Additional file 1.**

## Data Availability

The datasets used and/or analyzed during the current study are available from the corresponding author on reasonable request.

## Abbreviations

NPC: Nasopharyngeal carcinoma
CR: Complete response
PR: Partial response
IMRT: Intensity-modulated radiotherapy
MRI: Magnetic resonance imaging
CT: Computed tomography
PET/CT: Positron emission tomography computed tomography
EBV: Epstein–Barr virus
OS: Overall survival
HR: Hazard ratio
CI: Confidence interval
TI: The time interval between initial radical radiotherapy and metastasis
EM: Early metastatic
LM: Late metastatic
PCT: Palliative chemotherapy
ROC: Receiver operating characteristic
IQR: Interquartile range
LT: Local treatment
