# Supplementary material for: Clinical characteristics and prognostic factors affecting survival after radical radiotherapy for early and late post-treatment metastatic nasopharyngeal carcinoma
Source: BMC Cancer. 2023 Jan 3;23:7. doi: 10.1186/s12885-022-10494-7 (PMC9811739; doi:10.1186/s12885-022-10494-7)
Supplement: Supplementary file 1 — Additional file 1. [file 12885_2022_10494_MOESM1_ESM.docx]

| **Organ** | **Total (n=559)** | **EM group**  **(n=297)** | **LM group**  **(n=262)** |
| --- | --- | --- | --- |
| Liver | 97(17.4%) | 67(22.6%) | 30(11.5%) |
| Bone | 134(24.0%) | 81(27.3%) | 53(20.2%) |
| Lung | 114(20.4%) | 50(16.8%) | 64(24.4%) |
| Distant nodal | 19(3.4%) | 5(1.7%) | 14(5.3%) |
| Liver and bone | 44(7.9%) | 27(9.1%) | 17(6.5%) |
| Liver and lung | 21(3.8%) | 8(2.7%) | 13(5.0%) |
| Liver and distant nodal | 20(3.6%) | 8(2.7%) | 12(4.6%) |
| Bone and lung | 27(4.8%) | 13(4.4%) | 14(5.3%) |
| Bone and distant nodal | 16(2.9%) | 8(2.7%) | 8(3.1%) |
| Lung and distant nodal | 29(5.2%) | 10(3.4%) | 19(7.3%) |
| Liver, bone and lung | 10(1.8%) | 7(2.4%) | 3(1.1%) |
| Liver, bone and distant nodal | 7(1.3%) | 4(1.3%) | 3(1.1%) |
| Liver, lung and distant nodal | 6(1.1%) | 1(0.3%) | 5(1.9%) |
| Lung, bone and distant nodal | 3(0.5%) | 0(0.0%) | 3(1.1%) |
| Liver, bone, lung and distant nodal | 3(0.5%) | 2(0.7%) | 1(0.4%) |
| Others (breast, adrenal, spleen, brain) | 9(1.6%) | 6(2.0%) | 3(1.1%) |

**Table S1. Comparison of multiple organ metastases of EM and LM patients with metastatic NPC (n=559)**

Abbreviations: EM = early metastatic; LM=late metastatic.
